# Effect of acute pesticide exposure on bee spatial working memory using an analogue of the radial-arm maze

**DOI:** 10.1038/srep38957

**Published:** 2016-12-13

**Authors:** Elizabeth E. W. Samuelson, Zachary P. Chen-Wishart, Richard J. Gill, Ellouise Leadbeater

**Affiliations:** 1 School of Biological Sciences, Royal Holloway University of London, Egham, United Kingdom; 2 Department of Physics, Royal Holloway University of London, Egham, United Kingdom; 3 Department of Life Sciences, Imperial College London, Silwood Park campus, Ascot, United Kingdom

## Abstract

Pesticides, including neonicotinoids, typically target pest insects by being neurotoxic. Inadvertent exposure to foraging insect pollinators is usually sub-lethal, but may affect cognition. One cognitive trait, spatial working memory, may be important in avoiding previously-visited flowers and other spatial tasks such as navigation. To test this, we investigated the effect of acute thiamethoxam exposure on spatial working memory in the bumblebee *Bombus terrestris*, using an adaptation of the radial-arm maze (RAM). We first demonstrated that bumblebees use spatial working memory to solve the RAM by showing that untreated bees performed significantly better than would be expected if choices were random or governed by stereotyped visitation rules. We then exposed bees to either a high sub-lethal positive control thiamethoxam dose (2.5 ng^−1^ bee), or one of two low doses (0.377 or 0.091 ng^−1^) based on estimated field-realistic exposure. The high dose caused bees to make more and earlier spatial memory errors and take longer to complete the task than unexposed bees. For the low doses, the negative effects were smaller but statistically significant, and dependent on bee size. The spatial working memory impairment shown here has the potential to harm bees exposed to thiamethoxam, through possible impacts on foraging efficiency or homing.

Animal pollinators are key ecosystem service providers across most terrestrial landscapes. Ecologically, they pollinate around 87.5% of flowering plants worldwide^1^, and economically are responsible for approximately 9.5% of the value of global food production^2^. Insects represent the vast majority of animal pollinators^3^, with bees providing a large contribution to this pollination service^3^,^4^. It is therefore of concern that bees are considered to be under threat^4^,^5^, and understanding the stressors affecting bees and the mechanisms by which they impair bee behaviour and health is of ecological and economic importance^6^.

The application of pesticides in the environment, primarily for agriculture and horticulture, has been implicated as a principal threat to pollinating insects^7^. Whilst pesticides are used to target pest insect species, they may inadvertently harm visiting insect pollinators if applied to a flowering crop. For instance, neonicotinoids are a widely used class of agricultural insecticide^8^ that act systemically, allowing the pesticide to be transported to all parts of the plant including the nectar and pollen of flowers^9^,^10^. Insect pollinators foraging on these flowers can thus be exposed to neonicotinoid residues^11^. Although concentrations of neonicotinoids in the nectar and pollen of treated plants are unlikely to be high enough to be lethal to bees^12^, growing evidence is emerging for sub-lethal effects on bee behaviour and physiology^13^,^14^,^15^,^16^, which may lead to subsequent colony-level impacts^17^,^18^,^19^.

Neonicotinoids act as agonists of insect nicotinic acetylcholine receptors^20^, disrupting neural transmission and cognitive processes^14^,^15^. Correspondingly, olfactory learning and memory have been shown to be impaired by sub-lethal neonicotinoid exposure in honeybees^21^,^22^ and bumblebees^23^. However, the effects of pesticides on spatial learning and memory have been highlighted as an evidence gap, and standard assays to assess effects on behavioural processes such as spatial memory have been called for^24^. Spatial memory is extremely important to central place foragers, such as bees, both for navigation when returning to the nest after foraging trips^25^ and for increasing foraging efficiency^26^. Reduced homing ability of honeybees following acute sub-lethal doses of neonicotinoids has been demonstrated^27^,^28^,^29^, with evidence suggesting that impairment of spatial reference memory (e.g. long-term retention of landmarks^30^) could be responsible^29^. Spatial working memory, on the other hand, involves maintaining short-term task-relevant information^31^, such as remembering and avoiding flowers that have already been depleted in a foraging patch^32^,^33^, an efficient foraging strategy often employed by bees and other nectarivores^34^,^35^. Here, we investigate the effect of pesticide exposure on spatial working memory in a key pollinator, *Bombus terrestris* L. (Fig. 1a).

The radial-arm maze (RAM) was originally designed for use in rodents by Olton & Samuelson^36^ and is a well-established method for studying pharmacological effects on spatial working memory^37^. The task requires animals to identify and remember previously visited reward locations in order to avoid revisits^37^, making it particularly relevant to the foraging ecology of nectarivores^35^. Variations on the RAM have frequently been used in nectarivorous birds e.g.^33^; however, its potential in bees has been explored to only a limited extent in basic memory research^32^,^38^,^39^. Here we adapt this tool for application to bumblebee foraging, and apply it to study the impact of a stressor on insect cognition.

The aim of this experiment was to test the acute effects of thiamethoxam at concentrations that could be encountered in the field on spatial working memory in the bumblebee *B. terrestris.* Thiamethoxam is one of three neonicotinoid pesticides currently subject to an EU restriction regarding use on bee-attractive crops, for which a review is imminent^40^, and was the most widely used on flowering crops in the UK prior to the restriction^8^. We focus on the consequences of acute doses because these may be particularly severe in the context of spatial memory, where reduced foraging efficiency or failure of foragers to return to the nest could have immediate knock-on effects at the colony level^17^,^19^. We first validated our protocol by confirming that bumblebees use spatial memory to complete the RAM task efficiently (which requires bees to visit the eight presented flowers, minimizing revisits, and return to the nest), comparing initial performance of bees prior to pesticide treatment to simulated chance performance. We then tested for an effect of pesticide on RAM performance at a high dose (2.5 ng^−1^ bee) as a positive control, and two different low doses (LD.091: 0.091 ng^−1^ bee and LD.377: 0.377 ng^−1^ bee) based on estimates of field-realistic exposure.

## Results

### Protocol validation: comparison to chance and stereotypical simulations

Bees could complete a RAM by choosing locations to visit randomly, by using stereotyped movement rules (e.g. “always move clockwise”, “preferentially move upwards”), or by using spatial working memory. Analysis of the final training bout (i.e. prior to pesticide exposure) showed that bees displayed a moderate tendency to travel to the nearest neighbouring flower (“contiguity preference”), and in addition, transition frequency between neighbouring flower pairs was negatively correlated with angle from vertical between flowers (Spearman’s rho = −0.87) implying an additional preference to fly vertically upwards (see Supplementary Fig. S1). However, comparison of observed performance in this trial confirmed that individuals additionally use spatial working memory to complete the task. Observed performance (mean correct choices in the first eight visits, correct choices before first revisit and total revisits) on the final training bout for all bees was compared to simulated performance derived from a Monte Carlo simulation of the task run with 1,000,000 iterations. Bees performed better than both random transition (C) and chance plus stereotypical behaviour (C + S) on all three measures, as simulated means did not fall within the 95% CIs of the observed data (Fig. 2).

### Performance on the RAM following pesticide exposure

Sixty one bees were trained on the RAM, after which each was assigned to one of four pesticide doses: 0 ng (n = 16, “Control”), 0.091 ng (n = 14, “LD.091”), 0.377 ng (n = 16, “LD.377”) or 2.5 ng (n = 15, “ High”) and subjected to a final testing bout 45 minutes after exposure to test the effect of pesticide on RAM performance. Four measures of RAM performance were assessed: i) total revisits to previously visited flowers^37^, representing the number of spatial working memory errors made, ii) correct choices before first revisit^41^, iii) correct choices in the first eight visits^36^ and iv) time per visit as a measure of decision-making time.

#### Total revisits

The most parsimonious model for total revisits contained treatment, size, and their interaction term (Table 1a). In other words, pesticide treatment had a strong effect on the number of revisits bees made to flowers they had already depleted (Fig. 3a), but this depended upon the size of the bee (Fig. 4, Table 2a). To obtain individual effect size estimates for each dose that took into account bee size, we thus split the dataset into two groups: ‘smaller bees’ with a thorax width of ≤5.46 mm (n = 31; mean (range) = 5.21 (4.51–5.46)) and ‘large bees’ >5.46 mm (n = 30; mean (range) = 5.68 (5.48–6.07)), and repeated the analysis separately on the subset data. For the smaller bees there was no effect of treatment on total revisits (ΔAIC between treatment only model and basic model: −0.9). However, for large bees, there was a strong effect of treatment on total revisits (ΔAIC to basic model: 7.36). Both the High and LD.377 groups significantly differed from the Control (High: estimate = 1.032, 95% CIs = [0.517–1.547]; LD.377: estimate = 0.727 [0.147–1.307], indicating that pesticide exposure influenced bee performance even at field-realistic levels.

#### Correct choices before first revisit

The optimal Cox proportional hazards model included treatment as the only independent variable (Table 1b), implying that pesticide treatment lowered the number of choices bees made before revisiting a drained flower (Fig. 5). The high treatment level had a high hazard ratio (HR; a measure of effect size) with 95% CIs that do not include one (model averaged HR = 2.292 [1.060–4.959]), as did the LD.091 group (model averaged HR = 2.306 [1.092–4.866]), indicating a significant negative effect on the number of correct choices made before the first revisit (Table 2b). We found no evidence of an effect in the LD.377 group (model averaged HR = 1.366[0.666–2.800]). Note that we also found no evidence of a size-dependent effect (ΔAIC to model with interaction term = 5.11), so smaller and large bees were considered within the same analysis.

#### Time per visit and correct choices in the first eight visits

There was a trend towards fewer correct choices in the first eight visits in bees treated with the high pesticide dose (Fig. 3b). However, there was no significant effect of treatment on correct choices in the first eight visits as model selection found the basic model to be most parsimonious (Supplementary Table S1a). We also found no effect of treatment on time per visit (task duration divided by total revisits; Supplementary Table S1b), although size had a negative effect (estimate = −0.335 [−0.536–−0.135]).

## Discussion

Here, we have adapted the RAM to test the effect of a stressor on bumblebee spatial working memory for the first time. We first demonstrated that untreated bees performed better than chance simulations and better than would be expected based on stereotyped movement rules, implying that tested bees were indeed employing spatial working memory. Following this, we tested bees treated with a high dose of the neonicotinoid thiamethoxam as a positive control and observed negative effects on bumblebee spatial working memory. We then investigated how bees performed on the RAM after being treated with two different low doses based on estimated field-realistic exposure and we found small but detectable size-dependent effects on spatial working memory.

Acute exposure to the high, positive control dose of thiamethoxam (2.5 ng^−1^ bee) negatively affected two of the RAM performance measures assayed. Bees in this treatment group made fewer correct choices before the first revisit compared to the control, suggesting they were less able to remember which flowers they had already depleted. This may represent a simple reduction in spatial memory use or an accompanied greater reliance on a stereotypical-movement-based strategy, which would be consistent with the finding that pesticide-treated bees made their first revisit earlier in their visit sequence. This could be tested in future experiments by interrupting visits (analogous to confinement in the rodent RAM^37^) to disrupt stereotypical transition sequences^42^.

Depending on body size, the high pesticide treatment also increased the number of revisits to flowers that had already been depleted during the task. For bees in this treatment group, size had a positive relationship with total revisits, with large bees making up to five times more revisits than smaller bees. This suggests larger bees experienced a stronger effect from the pesticide at the high dose, possibly as a result of absorbing more pesticide in the 45 minute holding period, as larger bees empty their crops (i.e. transfer nectar to the midgut) at a faster rate^43^. While this is the first demonstration of pesticide-induced spatial working memory impairment in bees, these findings supplement previous laboratory research that found negative effects of neonicotinoids on olfactory learning and memory in honeybees^21^ and bumblebees^23^. A plausible mechanism for the cognitive impairments observed in our and other studies is the mode of action of neonicotinoids, which have been shown to disrupt the neurophysiological properties of Kenyon cells in the mushroom bodies of bee brains. These are involved in complex cognitive functions including associative learning and spatial orientation^14^,^15^.

We found no effect of treatment, however, on time per visit, suggesting that decision-making time was not affected by pesticide. Correct choices in the first eight visits was also not significantly affected by pesticide treatment; this measure is sensitive only to errors made early in the choice sequence suggesting that the working memory impairment experienced by treated bees primarily took the form of repeated errors later in the trial in the course of finding the last “missed” flowers^37^.

While it is of biological interest to understand the mechanisms by which sub-lethal doses of pesticides affect bee behaviour, it is crucial to investigate whether these effects are likely to be seen at levels encountered in the field in order to inform policy decisions^44^,^45^. We found evidence that spatial working memory was impaired in some measures of RAM performance at both low doses based on field-realistic exposure (LDs) tested. The lower LD.091 treatment resulted in bees making fewer flower choices before they made their first mistake, while the higher LD.377 treatment increased total revisits (spatial memory errors) in large but not smaller bees. Bumblebees exhibit intra-colony size variation^46^. This experiment used bees at the larger end of the spectrum and only the largest of these were affected, suggesting that in nature only a limited proportion of a colony may be vulnerable to some of the spatial memory impacts seen here. Bees in the size cohort that was found to be strongly affected by pesticide (thorax width >5.46 mm) make up on average 22% of a colony’s foragers, but contribute more to the foraging effort, bringing in 29% of daily forage^47^ (D. Goulson, unpublished data) making spatial working memory in this section of a colony’s workforce likely to disproportionately affect colony function. In addition, the positive relationship between size and crop-emptying rate^43^ may mean smaller bees are also affected after a longer amount of time post-exposure than our experiment allowed for.

Could such memory impairment to a cohort of workers have an impact on colony fitness? Spatial working memory is important for foraging efficiency in flower-visiting animals^32^,^35^; in bumblebees, increased re-visitation rates result in lower rates of nectar or pollen intake^48^. Previous research has demonstrated reduced foraging efficiency in terms of the quantity and frequency of pollen collected by pesticide-exposed bumblebees^13^,^17^,^49^,^50^, which could be explained by the spatial working memory impairment demonstrated in this study. Less efficient foraging by workers may have knock-on effects at the colony level^17^, where reduced daily nectar and pollen supply could restrict colony growth and even result in colony failure^19^. Indeed, a recent study found that bumblebee colonies placed in neonicotinoid-treated oilseed rape fields gained less weight than control colonies, which the authors suggest was due in part to reduced foraging efficiency^51^.

While the RAM apparatus used here simulates most closely within-patch foraging decisions, spatial working memory is also involved in components of bee navigation including vector memory or path integration^52^, impairment of which could lead to reduced homing ability and consequently loss of foragers from the colony^27^. Our finding that acute effects can be seen after just 45 minutes indicates forager loss is a realistic possibility, as homing failure may occur while a forager is still in the field following ingestion of contaminated nectar^11^. Acute neonicotinoid exposure has been reported to cause homing failure in honeybee foragers^27^,^28^,^29^, (but see Stanley *et al*.^50^) but whether this is a consequence of spatial working memory impairment is not yet clear. Longer-term spatial reference memory is also used in homing^30^, and a study by Fischer, *et al*.^29^ using honeybees found pesticide-induced disruption of this component of navigation. Our demonstration of impaired spatial working memory suggests that the shorter term, vector component of navigation could also be affected by exposure to neonicotinoids.

We have demonstrated that spatial working memory can be impaired just 45 minutes after a single dose of thiamethoxam. In real agricultural environments, however, bees are likely to be exposed to pesticides chronically over several weeks, typically during the bloom period of mass-flowering crops such as oilseed rape^13^,^51^. Evidence suggests that sub-lethal impacts on individuals^23^ and colonies^19^ may be more severe following chronic, compared to acute, exposure to pesticides. However, a response following acute exposure does not necessarily predict the presence or magnitude of chronic effects, due to mediating factors such as the rate at which bees clear pesticide from the body^53^. As such, future research should investigate whether the acute effects found here may be magnified with chronic exposure^13^. Alone or combined with other known sub-lethal effects^54^, spatial working memory impairment has the potential to harm bees that are exposed to thiamethoxam. With review of the scientific evidence for the EU neonicotinoid restriction imminent^40^, it is important that policy makers consider all potential impacts of sub-lethal effects on bee populations.

## Materials and Methods

### Bees

We obtained seven commercial *B. t. audax* colonies from Koppert Ltd, Haverhill, UK. Each colony was transferred to a bipartite wooden nest box on arrival, which was connected to a foraging arena (0.5 m × 0.5 m × 0.5 m) by a transparent Perspex tube containing four sliding ‘trap-doors’ to control the flow of bees entering and returning from the arena. When not being trained or tested, bees were allowed unrestricted access to the arena and provided with 43% (w/w) sucrose solution (see Supplementary Methods) *ad libitum* from two gravity feeders located on the back wall of the arenas in the centre of the artificial flower array (see later section for details on the array). Thirty grams of pollen (in the form of honeybee collected pellets, supplied by Koppert Ltd, Haverhill, UK) were provided every 2–3 days directly into the nest box. Thorax width (between tegulas (wing joints)) of all tested bees was measured following freezing as the mean of three digital calliper measurements (accuracy = 0.01 mm).

### Radial-Arm Maze Apparatus

**Figure 1:**
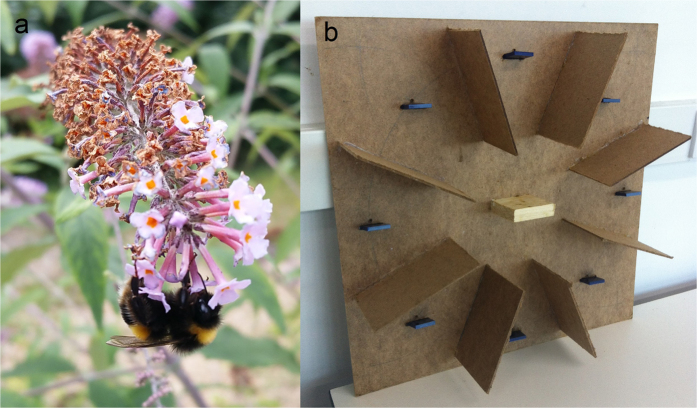
(**a**) A foraging *Bombus terrestris* L. worker. To maximise nectar intake, a forager must avoid revisiting flowers it has already depleted. *Photo credit:* Andres Arce. (**b**) Radial-arm maze apparatus adapted for bumblebees. The apparatus consists of a circular array of eight artificial flowers on a vertical board with 10 × 15 cm wooden baffles between each flower. Two gravity feeders containing sucrose solution were provided on the central platform between training sessions.

**Figure 2:**
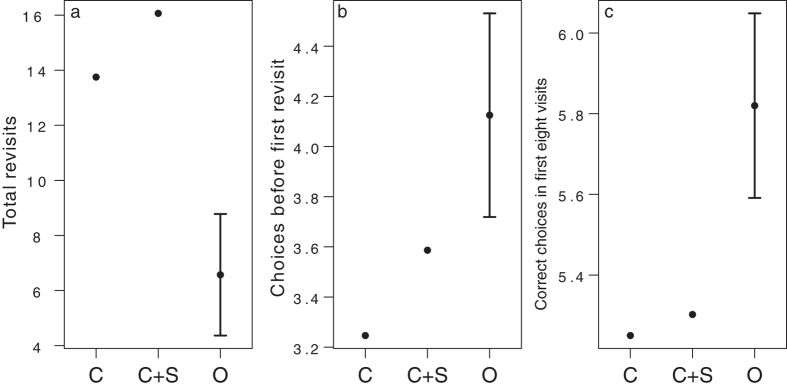
Comparison of observed bumblebee performance on the radial-arm maze with simulated performance. Means generated from Monte Carlo simulations simulating pure chance performance (C) and chance + stereotypical behaviour performance (C + S) compared to observed means and 95% confidence intervals for all bees before pesticide treatment (O) for (**a**) total revisits (n = 30), (**b**) choices before first revisit (n = 61) and (**c**) correct choices in the first eight visits (n = 61). Observed data for total revisits include only bees that visited all eight flowers. Before pesticide exposure bees performed better than both C and C + S in all three measures, implying the use of spatial memory to complete the task.

**Figure 3:**
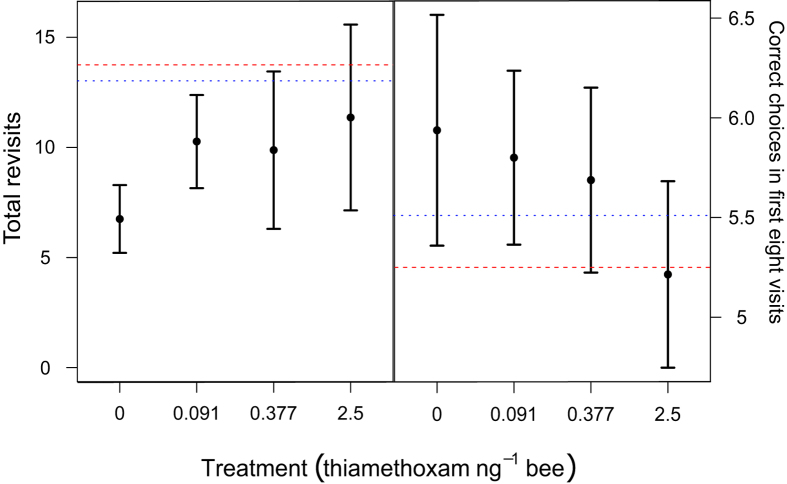
Total revisits and correct choices in the first eight visits on the radial-arm maze following acute pesticide exposure. Mean (**a**) total revisits and (**b**) correct choices in the first eight visits with 95% confidence intervals for bees treated with one of four pesticide treatments: 0 ng per bee (control, n = 16), 0.091 ng per bee (LD.091, n = 14), 0.377 ng per bee (LD.377, n = 16) and 2.5 ng per bee (high, n = 15). Horizontal lines indicate simulated performance when bees behave at random (red dashed) and using stereotypical behaviour only (blue dots).

**Figure 4:**
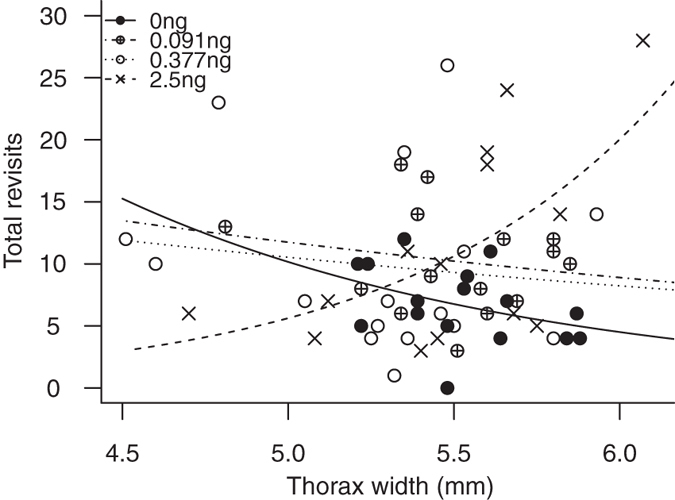
Scatterplot of total revisits against bee size (thorax width) grouped by treatment (ng per bee), showing an interaction between treatment and size for 61 bees exposed to four different pesticide treatments. Lines for each treatment are predicted from a negative binomial GLMM including the treatment*size interaction.

**Figure 5:**
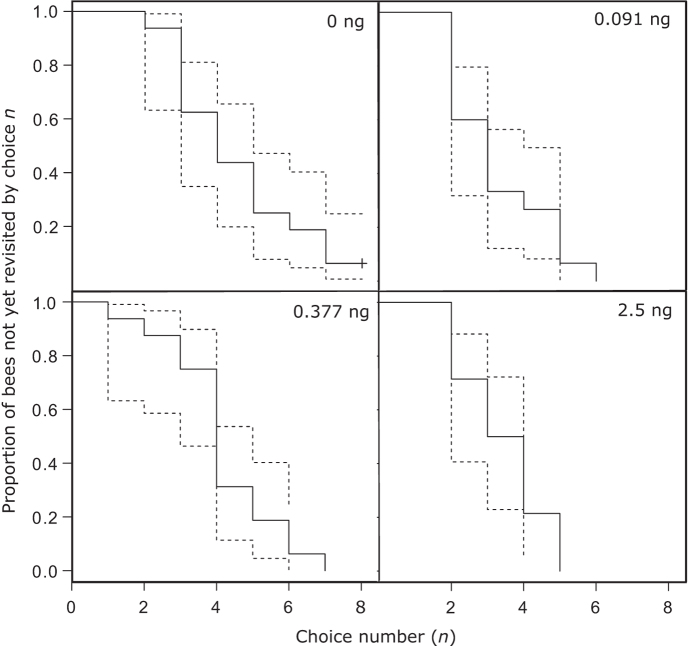
Kaplan-Meier curves of choices before first revisit for four pesticide treatments. Each step represents the choice at which a bee or bees made their first revisit; for example, all bees in the high group made a revisit by choice five, while all but one bee in the control group made a revisit by choice eight. Dashed lines indicate standard errors.

A typical RAM consists of eight arms, or reward locations; an animal must remember which arm(s) it has already visited to avoid revisiting a depleted location^36^. Adaptation of the RAM for flying animals (e.g. birds) typically applies an “open field” approach where all reward locations can be viewed from the centre of the array but no enclosed arms or central chamber are present^33^,^55^,^56^. In bumblebees, foraging within a patch typically involves flying between flowers or inflorescences, so a RAM apparatus that requires bees to fly represents a more ecologically relevant foraging decision than one where bees walk through maze arms. To adapt the RAM for bumblebees we built a circular array of eight artificial flowers (hereafter ‘flowers’) on a vertical board that constituted the back wall of the experimental arena. Flowers consisted of three square plastic chips glued together, which could be inserted through slots in the board (Fig. 1b.) The blue chip on the arena side of the board acted as a landing platform from which a bee could obtain a reward (a 10 μl droplet of 43% (w/w) sucrose solution) by inserting its proboscis through a 4 mm hole above the slot to reach the chip on the other side. Flowers were immediately replaced with clean ones after a bee had fed (while feeding on the subsequent flower), to remove information about visitation from scent marks^57^,^58^.

Wooden baffles (dimensions: 10 × 15 × 0.3 cm) were positioned between each flower so that bees had to fly to the centre of the arena to see the entire maze, as in the rodent RAM^36^, minimising the use of stereotypical movement rules such as travelling from one flower to the nearest neighbouring flower and preventing bees from seeing any other flowers when on a particular flower, meaning that each represented an independent reward location (see Supplementary Methods).

### Group Training

**Figure 6:**
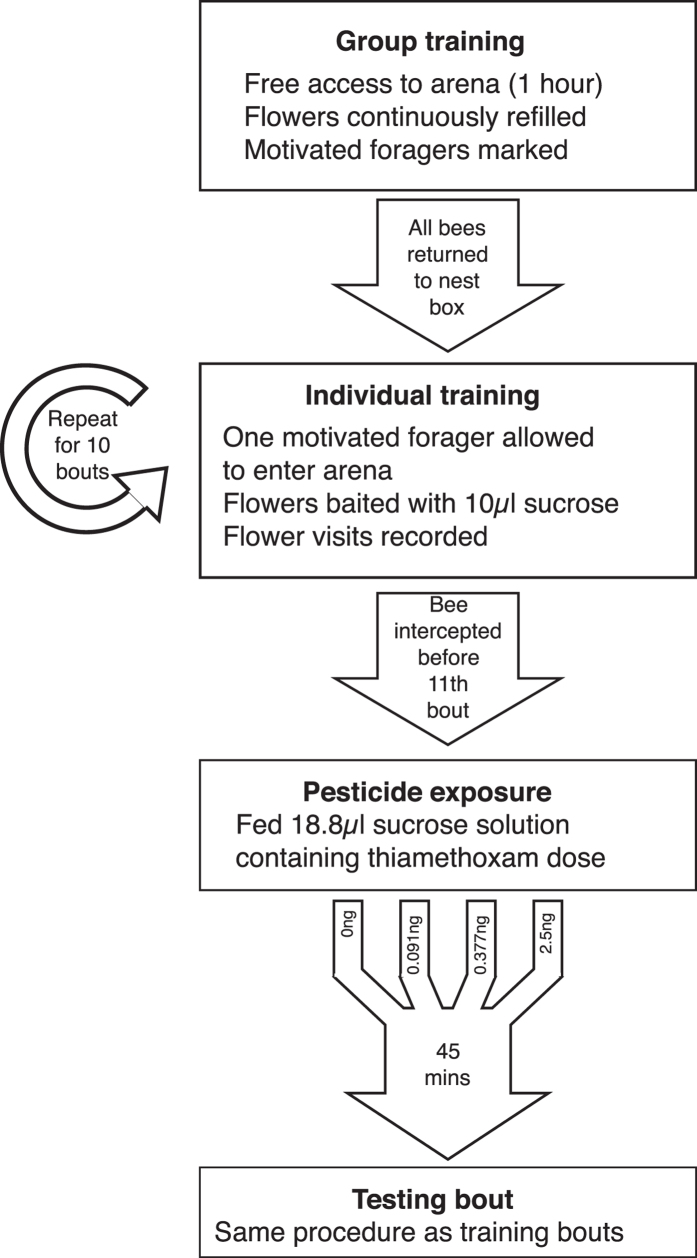
Overview of the methods used to test pesticide treated bees on the radial-arm maze.

Prior to testing, an hour-long group training period was allowed each day to train bees to use the artificial flowers and to identify motivated foragers (Fig. 6). Feeders were removed from the arena and the flowers were loaded with sucrose solution, which was continuously refilled during the group training session. Bees were allowed free access to the arena and those that spontaneously learnt to feed from the flowers during this period were tagged with a unique numbered disc (Opalith Plättchen, Christian Graze KG, Germany) and returned to the colony. If a feeding bee had already been marked from a previous training session its identity was recorded. After one hour all bees in the arena were returned to the nest box.

### Individual Training

The RAM task required bees to visit all eight flowers and return to the nest. The first bee to arrive at the tunnel entrance that had been marked or recorded feeding in that morning’s group training session was chosen as a test subject, and allowed to enter the arena alone and feed from the flowers which had been reloaded with sucrose solution; this time flowers were not refilled after visitation and were instead replaced with clean, empty flowers to remove scent information. Visits to flowers were recorded as either ‘correct’ (flowers that had not yet been visited) or ‘revisits’ (to flowers that had been depleted). ‘Land’, where a bee landed on the platform but did not feed, and ‘approach’, where a bee came within 1 cm of the platform or hole, was also recorded. Flower visits were recorded as soon as the bee inserted its proboscis through the hole. After visiting the flowers the bee was permitted to return to the colony to empty its crop (see Supplementary Methods). The duration of the period in the arena, defined as a foraging bout, was recorded. Bees were given ten training bouts on the maze before pesticide exposure (data from a pilot study showed that the majority of bees reach asymptotic performance on the maze after ten bouts; see Supplementary Material).

### Pesticide Exposure and Testing

Acute thiamethoxam doses were calculated to simulate the total pesticide consumed by a bee foraging for one hour on oilseed rape nectar contaminated with thiamethoxam at two concentrations over a range that can be found in the field: 2.4ppb, based on residues found in *B. terrestris* nectar pots^59^ and oilseed rape nectar in honeybee crops^60^ and 10ppb, based on residues in nectar of treated plants^12^,^61^. This equated to 0.091 ng of active ingredient per bee at a 2.4ppb concentration and 0.377 ng at 10ppb (for dose calculations see Supplementary Methods). An additional high dose of 2.5 ng per bee (half the acute oral toxicity LD50 for honeybees^45^) was used to test for effects at non-field-realistic levels, as a positive control.

After ten training bouts each bee was assigned to one of the four pesticide exposures: 0 ng (n = 16, “Control”), 0.091 ng (n = 14, “LD.091”), 0.377 ng (n = 16, “LD.377”) or 2.5 ng (n = 15, “High”). The bee was intercepted on its way to the arena and fed 18.85 μl of sucrose solution containing the relevant dose of pesticide (see Supplementary Methods), after which it was returned to the tunnel where it was held for 45 minutes after exposure to maximise pesticide absorption^27^ but avoid a decline in motivation. This represents a conservative estimate of the effects as the full dose of pesticide may take longer than this to be absorbed^43^ or there may be a time lag between absorption and effects being seen^62^, as thiamethoxam causes functional responses only after metabolism to clothianidin^63^. Following this the bee was allowed back into the arena for the final testing bout, which followed the same procedure as the training bouts.

### Simulation of chance performance

To confirm that bees use spatial working memory to complete the RAM, observed performance (total revisits, choices before the first revisit and correct choices in the first eight visits) on the final training bout for all bees was first compared to chance performance derived from a Monte Carlo simulation of the task run with 1,000,000 iterations (see Supplementary Fig. S3). The chance simulation assumed all flowers were visited with equal probability, so if observed error rate was no less than that obtained from simulated chance performance it would indicate that bees visited flowers at random without using spatial memory.

Animals may also employ stereotypical movement rules, such as travelling to the nearest neighbouring flower, which can increase RAM performance without the use of spatial memory^37^. The RAM simulates within-patch foraging decisions, a context in which bees are known to employ heuristic movement rules in addition to spatial memory^64^. To account for this, we ran a second Monte Carlo simulation in which simulated bees did not move between flowers at random, but chose their next target flower according to a transition probability distribution (see Supplementary Fig. S1) obtained from pooled choices made during all final training bouts by actual bees in the experiment^38^. For example, a simulated bee visiting flower 1 would have a 19% chance of visiting flower 2 next, because the real bees moved to flower 2 on 19% of occasions, but importantly, whether the target flower had already been visited or not did not influence the simulated bees’ decisions. If observed performance was no different to this simulation, it could be concluded that performance was only explained by stereotypical behaviour. On the other hand, if observed performance was better than both the chance and stereotypical simulations it could be assumed spatial memory was being employed for the task in addition to stereotypical behaviour.

### Statistical analysis

For each of the four assessed measures of RAM performance, a set of candidate models were fitted. We used an “all-subset” approach, with the full model containing bee size (continuous) and treatment (categorical), plus their interaction, as fixed effects. The comparison set included all subsets of this model, including the basic model containing only the constant and residual variance. Each candidate model also contained “colony” as a random effect. We selected the model or set of models with the lowest Akaike Information Criteria (AIC) as the best fitting model(s)^65^. Where several models were within two AIC units of the best model, model averaging was carried out to obtain parameter estimates derived from the best set of models including the basic model if applicable^66^. Final models were validated graphically to assess fit and check that assumptions had been met^67^.

For total revisits, a negative binomial GLMM (to account for over-dispersion) with log link and colony as a random effect was used. Correct choices in the first eight visits was analysed by considering each choice in the first eight visits as a binary response (success or failure) and using a binomial GLMM with logit link and bee nested within colony as random effects. Time per visit (log-transformed task duration divided by total visits) was analysed using linear mixed models with colony as a random effect. Correct choices before first revisit was subjected to a survival analysis using non-parametric Cox proportional hazards models. All analyses were conducted in R version 3.2.1^68^ (for packages see Supplementary Methods).

## Additional Information

**How to cite this article:** Samuelson, E. E. W. *et al*. Effect of acute pesticide exposure on bee spatial working memory using an analogue of the radial-arm maze. *Sci. Rep.* **6**, 38957; doi: 10.1038/srep38957 (2016).

**Publisher’s note:** Springer Nature remains neutral with regard to jurisdictional claims in published maps and institutional affiliations.

## Supplementary Material

Supporting Information

## Figures and Tables

**Table 1:** Tables of candidate models (a) using negative binomial GLMMs to investigate the effect of pesticide treatment and bee size on total revisits and (b) using Cox proportional hazards models to investigate the effect of pesticide treatment and bee size on choices before first revisit.

Model	AIC	ΔAIC	*w*_*i*_
a	Total revisits		
Treatment * size	**378.9**	**0**	**0.527**
Treatment	381	2.18	0.177
Basic	381.2	2.36	0.162
Treatment + size	382.9	4.03	0.07
Size	383.1	4.22	0.064
**b**	Choices before first revisit		
**Treatment**	**384.7**	**0**	**0.41**
**Basic**	**385.5**	**0.79**	**0.276**
**Treatment + size**	**386.5**	**1.81**	**0.166**
Size	387.2	2.51	0.117
Treatment * size	389.8	5.11	0.032

In all cases, the basic model included the constant and the residual variance, with all other models containing the basic model plus the indicated covariates. Models are presented in order of ΔAIC from the best model alongside their respective Akaike weights (*w*_*i*_). The best sets of models which were averaged to obtain model averaged estimates (models <2 ΔAIC from the model with the lowest AIC) are highlighted in bold.

**Table 2:** Coefficients and 95% confidence intervals (CIs) for the optimal model or model sets to predict a) total revisits (treatment*size model with colony as a random effect) and b) correct choices before first revisit (model-averaged coefficients for treatment only, treatment + size and basic models.

Parameters					
a	Total revisits				
	Estimate	Std. Error	95% CIs		
			Lower	Upper	
(Intercept)	6.384	3.769	−1.003	13.771	
**Treatment (High)**	**−11.010**	**4.471**	**−19.773**	**−2.247**	
Treatment (LD.377)	−2.796	4.164	−10.957	5.365	
Treatment (LD.091)	−2.534	4.698	−11.742	6.674	
Size	−0.813	0.684	−2.154	0.528	
**Size:Treatment (High)**	**2.084**	**0.810**	**0.496**	**3.672**	
Size:Treatment (LD.377)	0.566	0.762	−0.928	2.060	
Size:Treatment (LD.091)	0.536	0.854	−1.138	2.210	
**b**	**Correct choices before first revisit**				
**Parameters**	**Regression coefficient (b)**	**Std. Error (SE(b))**	**Hazard Ratio (e^b^)**	**95% CIs on Hazard Ratio**	
				Lower	Upper
**Treatment (High)**	**0.8295**	**0.3937**	**2.292**	**1.060**	**4.959**
Treatment (LD.377)	0.3116	0.3663	1.366	0.666	2.800
**Treatment (LD.091)**	**0.8353**	**0.3811**	**2.306**	**1.092**	**4.866**
Size	0.1964	0.452	1.217	0.502	2.952

Parameters highlighted in bold are considered important to the model based on 95% CIs.
