# Supplementary material for: Effect of acute pesticide exposure on bee spatial working memory using an analogue of the radial-arm maze
Source: Sci Rep. 2016 Dec 13;6:38957. doi: 10.1038/srep38957 (PMC5154185; doi:10.1038/srep38957)
Supplement: Supporting Information [file srep38957-s1.pdf]

Supporting Information

Effect of acute pesticide exposure on bee spatial working memory using an analogue of the radial-arm maze

Elizabeth E. W. Samuelson[a*], Zachary P. Chen-Wishart[b], Richard J. Gill[c] & Ellouise Leadbeater[a]

[a] School of Biological Sciences, Royal Holloway University of London, Egham, United Kingdom

[b] Department of Physics, Royal Holloway University of London, Egham, United Kingdom

[c] Department of Life Sciences, Imperial College London, Silwood Park campus, Ascot, United Kingdom

*Corresponding author: elizabeth.samuelson.2014@live.rhul.ac.uk

Supporting Methods

Pesticide treatment

Thiamethoxam doses were calculated to simulate a bee foraging for one hour on oilseed rape nectar contaminated with thiamethoxam at two concentrations over a range that can be found in the field: 2.4ppb, based on residues found in *B. terrestris* nectar pots (Thompson *et al.* 2013) and oilseed rape nectar in honeybee crops (Pilling *et al.* 2013) and 10ppb, based on residues in nectar of treated plants (Pohorecka *et al.* 2012; Sanchez-Bayo & Goka 2014). A foraging worker must consume 0.54 calories per minute (Heinrich 1979), so 32.4 calories are required to sustain a foraging trip of one hour. The mean sugar content of 'Samourai' oilseed rape nectar is 21.5% (Pierre *et al.* 1999), and sugar contains approximately 4cal mg^{-1} (Heinrich 1979). The amount of nectar (in mg) consumed for one hour of foraging can be calculated as calories required/(sugar content x cal mg^{-1} of sugar), so a bee would need

to ingest 37.7mg (≈µl) of the nectar collected during an hour on oilseed rape. This equates to 0.091ng of active ingredient per bee at a 2.4ppb concentration and 0.377ng at 10ppb. This is within the range estimated for one hour of foraging in honeybees (at 10-40% sugar concentrations; EFSA 2012).

Feeding 37.7µl of sucrose solution to a bee prior to the task is likely to lower motivation, so this volume was halved and the concentration of sugar and pesticide doubled to keep the amount of active ingredient received by each bee the same. Bees were therefore fed 18.85µl of 43% (w/w) sucrose solution containing either 0ppb (control), 4.8ppb (0.091ng per bee), 20ppb (0.377 ng per bee) and 133ppb (2.5 ng per bee) thiamethoxam. As in previous studies into acute effects of pesticides, the full dose was provided in one feed (Henry *et al.* 2012; Stanley, Smith & Raine 2015).

To obtain the required concentrations, we dissolved 100mg thiamethoxam ($C_8H_{10}ClN_5O_3S$ powder; PESTANAL® analytical standard, Sigma Aldrich, Poole, UK) in 100ml distilled water to produce a 1 ppt stock solution. Each fortnight aliquots of 1.2µl, 5µl and 33.3µl of the stock solution were diluted with 250ml 43% Brix sucrose solution to produce 4.8ppb, 20ppb and 133ppb thiamethoxam solutions respectively.

Radial Arm Maze (RAM) design

The RAM tests working spatial memory by requiring animals to remember which reward locations they have visited and avoid revisits (Foreman & Ermakova 1998). The original RAM was designed for rodents, for which a central chamber is appropriate to prevent animals moving from one reward location to the next in a circle; i.e. it reduces (but not eliminates) the use of stereotypical behaviour so that spatial working memory can be better identified. However, this approach is not perfectly suited to all animals; in particular, flying animals may behave unnaturally in an enclosed arm set-up. As such, previous studies on birds have used "open field" versions of the RAM to mimic a more natural setting, in which no central chamber is used (Balda & Kamil 1988; Hilton & Krebs 1990; Healy & Hurly 1995); our design refers to this approach. Bumblebee within-patch foraging typically involves flying between flowers/inflorescences (Pyke & Cartar 1992), so our RAM apparatus that requires bees to fly attempts

to represent a more ecologically relevant foraging decision than one where bees walk through maze arms.

The baffles between flowers fulfil a similar role to the rodent RAM central chamber in greatly reducing but not eliminating stereotypical behaviour (bees can fly over baffles, but cannot move directly from flower to flower), and preventing bees from seeing any other flowers when on a particular flower, meaning that each represents an independent reward location (like arms of the rodent RAM) and that bees fly out of the array to see and subsequently visit the other flowers, to some degree mimicking the return from an arm to the centre of the rodent RAM (Olton & Samuelson 1976). The apparatus was located in a cue-rich laboratory environment with constant lighting during testing; cues such as arena walls and baffles did not differ between treatments and no additional landmarks were provided in the foraging arena as cue use was not being explicitly tested.

Behaviour on the RAM

The vast majority of the time, bees fed on the full 10µl of sucrose solution once the proboscis made contact. On the rare occasions some solution remained, the flower was exchanged for a clean, empty one as usual once the bee had left. A revisit to the most recent flower visited was only counted if >20 seconds of flying occurred between visits. Exchanging visited flowers for clean ones was done from the back of the maze while the bee was feeding on its next flower to minimise disturbance.

For the first bout, 20µl droplets of sucrose solution were used to increase motivation; for the subsequent nine training bouts and the final testing bout 10µl drops were used to ensure all flowers could be visited before satiation. On the last flower, the experimenter increased the size of the drop to allow the bee to fill its crop and return to the colony (Burmeister, Couvillon & Bitterman 1995).

Access to the nest box was blocked while a bee was in the arena to encourage it to visit all eight flowers; however, if the bee made three attempts to return before visiting all eight flowers the entrance was unblocked and the bee allowed to return to the nest box to minimise loss of motivation. The entrance was also unblocked after a bee had been in the arena for longer than 20 minutes; if 30 minutes elapsed

the bee was guided towards the entrance with a plastic pot. In the final bout, recording of visits was stopped after 30 minutes, as a longer period in the maze may not be an accurate test of working memory.

To administer the pesticide dose after the tenth bout, the bee was intercepted on its way to the arena by manipulating the tunnel doors to direct the bee into a plastic pot (diameter: 60mm). After allowing the bee to acclimatise for approximately one minute, 18.85µl of sucrose solution containing the relevant dose of pesticide was fed to the bee using a pipette inserted through a hole in the pot.

Statistical analysis

We employed the AIC-IT approach as this does not only test the support for the null hypothesis but also the plausibility of the alternative hypothesis or hypotheses, making it appropriate for use in controlled experiments as well as observational studies (Lukacs *et al.* 2007; Richards, Whittingham & Stephens 2011). In addition, we view the AIC-IT approach as providing further advantages over the Fisherian approach because in cases where two or more models are almost as good (have similar AIC values), it is possible to perform model averaging to get a more accurate estimate of effect size based on weighted support (Johnson & Omland 2004). Quoting effect sizes and confidence intervals rather than p-values is based on current recommended practice (Nakagawa & Cuthill 2007; Halsey *et al.* 2015).

AIC was chosen over AICc (corrected AIC for small sample sizes) as AICc can be overly conservative and has been shown to have little advantage over AIC (Richards 2005; Raffalovich *et al.* 2008). The R packages glmmADMB (Skaug *et al.* 2014), survival (Therneau 2015), MuMIn (Barton 2015), plotrix (Lemon 2006) and lattice (Sarkar 2008) were used for model fitting and producing graphs.

Pilot study

Before the experiment began, 14 bees from a separate colony were tested on the RAM to confirm that bees would interact with the maze and feed from the artificial flowers and to ascertain the number of training bouts needed before asymptotic performance was reached. Ten training bouts were used because the data for both correct choices in first the eight choices and total revisits showed performance reached an asymptote by bout ten (Fig. S2).

Six bees were also tested on a partially baited version of the maze to ensure bees were not able to detect the presence of a reward behind the flowers (e.g. by olfaction). Four flowers were randomly selected to be baited and the first four unique (non-repeated) flower visits recorded. Mean number of visits to baited flowers in the first four unique choices was 1.83, which was not significantly different from the chance expectation of 2 (one-sample t-test, t=-0.30715, df=5, p=0.7711).

Supporting Results and Discussion

Repeating the analysis excluding bees that did not visit all eight flowers (n=13) did not affect the outcome so the final analysis includes all 61 bees.

Total revisits

Analysing the full dataset but omitting the positive control still showed an effect on total revisits at field-realistic doses. Removing the high dose reduced support for the model containing a size * treatment interaction but increased support for the other models including treatment (Table S2). Model averaged effect size estimates (MAE) showed a significant difference between LD.091 and the control (MAE = 0.417, 95% CIs = [0.027– 0.807], Table S3), indicating that this field-realistic dose increased total revists.

One observation included a bee in the high treatment group with thorax width 6.07mm and 28 total revisits. To examine the effect of this observation on the treatment * size interaction found for total revisits, we repeated the analysis without this data point. Removing the observation reduced support for the model containing the treatment * size interaction (ΔAIC to best model: 2.84) although the treatment * size interaction at the high treatment level was still significant in that model (estimate = 2.291, 95% CIs = [0.311 – 4.271]) . Model averaging of the new best model set (treatment, size and basic) showed a significant positive effect of treatment on total revisits in the high dose (model averaged estimate = 0.434, 95% CIs = [0.001 – 0.867]), despite the highest total revisits value having been omitted.

Correct choices before first revisit

Analysing the data without the positive control did not change the outcome, with the model containing treatment only having the lowest AIC (ΔAIC to next best model = 2) with the hazard ratio (HR) for LD.091 positive and significantly different to the control (HR = 2.276, 95% CIs = [1.077– 4.810], Table S3).

Monte Carlo simulation and behaviour on the RAM

Holding the bee in the tunnel for 45 minutes following pesticide exposure did not visibly decrease motivation (bees flew directly to the array on entering the arena) and had no effect on maze performance (no difference between final training bout and testing bout in control bees for total revisits ($t = 0.458$, $df = 27.0$, $p = 0.651$), correct choices in the first eight choices ($t = -1.0132$, $df = 27.908$, $p = 0.3197$) and correct choices before first revisit ($t = -1.395$, $df = 29.5$, $p = 0.174$)).

The Monte Carlo simulations may overestimate total revisits as choices are made continuously until all eight flowers have been visited, whereas bees have physical limitations which are likely to restrict the total number of choices that can be made. To assess this, a conservative upper limit for number of revisits (18; two standard deviations above the observed mean) was applied to both the observed and C+S simulation data and the means recalculated. The simulated means remained outside the 95% CIs of the observed data (C = 9.94, C+S = 9.95, observed = 5.14[3.84-6.45]). Only bouts where all eight flowers were visited were included in the total revisits analysis (n=30). Every arrival at a flower ("visit", "revisit", "land" and "approach") was included in the transition probability matrix (Fig. S1a).

Stereotypical behaviour improved simulated RAM performance in terms of correct choices before the first revisit and correct choices in the first eight choices (see Fig. 5 in main text), due in part to the moderate contiguity ("nearest neighbour") preference and a low probability of revisiting the flower last visited. However, stereotypical behaviour increased total revisits; the most likely explanation for this is the unequal total frequencies with which each flower was visited (Fig. S1a). The contiguity preference and tendency to travel upwards is consistent with known bumblebee foraging behaviour (Pyke 1978).

Interestingly, bumblebee RAM performance (mean correct choices in the first eight choices (C) = 5.9) was not as high as in rats, for whom the maze was designed (C = 7.6; Bond, Cook & Lamb 1981), or other vertebrates including blue tits (C = 6.8; Hilton & Krebs 1990), *Betta splendens* (C = 6.6; Roitblat, Tham & Golub 1982) and pigeons (C = 6.3; Bond, Cook & Lamb 1981). This comparatively low but better-than-chance performance is consistent with two previous studies using an analogue of the RAM in honeybees (Brown & Demas 1994; Brown *et al.* 1997).

The success of applying our modified RAM to bumblebees is likely due to their ecological requirements as nectarivores, as the apparatus has been shown to be most relevant in species for whom the test mimics natural foraging ecology (Bond, Cook & Lamb 1981; Sulikowski & Burke 2010). Furthermore, bees perform a repeated foraging bout behaviour that can be exploited for this task. As such there is considerable potential for the application of this established lab technique in the study of the quantitative effects of pesticides and other stressors such as parasites on bumblebee cognition and learning behaviours.

References

Balda, R.P. & Kamil, A. (1988) The spatial memory of Clark's nutcrackers (Nucifraga columbiana) in an analogue of the radial arm maze. *Animal Learning & Behavior,* **16,** 116-122.

Barton, K. (2015) MuMIn: Multi-Model Inference.

Bond, A.B., Cook, R.G. & Lamb, M.R. (1981) Spatial memory and the performance of rats and pigeons in the radial-arm maze. *Animal Learning & Behavior,* **9,** 575-580.

Brown, M.F. & Demas, G.E. (1994) Evidence for spatial working memory in honeybees (Apis mellifera). *Journal of Comparative Psychology,* **108,** 344-352.

Brown, M.F., Moore, J.A., Brown, C.H. & Langheld, K.D. (1997) The existence and extent of spatial working memory ability in honeybees. *Animal Learning & Behavior,* **25,** 473-484.

Burmeister, S., Couvillon, P. & Bitterman, M. (1995) Performance of honeybees in analogues of the rodent radial maze. *Animal Learning & Behavior,* **23,** 369-375.

EFSA (2012) Statement on the findings in recent studies investigating sub-lethal effects in bees of some neonicotinoids in consideration of the uses currently authorised in Europe. *EFSA Journal,* **10,** 27.

Foreman, N. & Ermakova, I. (1998) The Radial Arm Maze: Twenty Years On. *A Handbook of Spatial Research Paradigms and Methodologies* (eds N. Foreman & R. Gillett). Psychology Press, Hove.

Halsey, L.G., Curran-Everett, D., Vowler, S.L. & Drummond, G.B. (2015) The fickle P value generates irreproducible results. *nature methods,* **12,** 179-185.

Healy, S. & Hurly, T. (1995) Spatial memory in rufous hummingbirds (Selasphorus rufus): a field test. *Animal Learning & Behavior,* **23,** 63-68.

Heinrich, B. (1979) *Bumblebee Economics,* 1st edn. Harvard University Press.

Henry, M., Beguin, M., Requier, F., Rollin, O., Odoux, J.-F., Aupinel, P., Aptel, J., Tchamitchian, S. & Decourtye, A. (2012) A common pesticide decreases foraging success and survival in honey bees. *Science,* **336,** 348-350.

Hilton, S.C. & Krebs, J.K. (1990) Spatial memory of four species of Parus: performance in an open-field analogue of a radial maze. *The Quarterly Journal of Experimental Psychology,* **42,** 345-368.

Johnson, J.B. & Omland, K.S. (2004) Model selection in ecology and evolution. *Trends in ecology & evolution,* **19,** 101-108.

Lemon, J. (2006) Plotrix: a package in the red light district of R. *R-News,* **6,** 8-12.

Lukacs, P.M., Thompson, W.L., Kendall, W.L., Gould, W.R., DOHERTY, P.F., Burnham, K.P. & Anderson, D.R. (2007) Concerns regarding a call for pluralism of information theory and hypothesis testing. *Journal of Applied Ecology,* **44,** 456-460.

Nakagawa, S. & Cuthill, I.C. (2007) Effect size, confidence interval and statistical significance: a practical guide for biologists. *Biological Reviews,* **82,** 591-605.

Olton, D.S. & Samuelson, R.J. (1976) Remembrance of places passed: Spatial memory in rats. *Journal of Experimental Psychology: Animal Behavior Processes,* **2,** 97.

Pierre, J., Mesquida, J., Marilleau, R., Pham-Delègue, M. & Renard, M. (1999) Nectar secretion in winter oilseed rape, Brassica napus—quantitative and qualitative variability among 71 genotypes. *Plant Breeding,* **118,** 471-476.

Pilling, E., Campbell, P., Coulson, M., Ruddle, N. & Tornier, I. (2013) A Four-Year Field Program Investigating Long-Term Effects of Repeated Exposure of Honey Bee Colonies to Flowering Crops Treated with Thiamethoxam. *PloS one,* **8,** e77193.

Pohorecka, K., Skubida, P., Miszczak, A., Semkiw, P., Sikorski, P., Zagibajło, K., Teper, D., Kołtowski, Z., Skubida, M. & Zdańska, D. (2012) Residues of neonicotinoid insecticides in bee collected plant materials from oilseed rape crops and their effect on bee colonies. *Journal of Apicultural Science,* **56,** 115-134.

Pyke, G. & Cartar, R. (1992) The flight directionality of bumblebees: do they remember where they came from? *Oikos***,** 321-327.

Pyke, G.H. (1978) Optimal foraging in bumblebees and coevolution with their plants. *Oecologia,* **36,** 281-293.

Raffalovich, L.E., Deane, G.D., Armstrong, D. & Tsao, H.-S. (2008) Model selection procedures in social research: Monte-Carlo simulation results. *Journal of Applied Statistics,* **35,** 1093-1114.

Richards, S.A. (2005) Testing ecological theory using the information-theoretic approach: examples and cautionary results. *Ecology,* **86,** 2805-2814.

Richards, S.A., Whittingham, M.J. & Stephens, P.A. (2011) Model selection and model averaging in behavioural ecology: the utility of the IT-AIC framework. *Behavioral Ecology and Sociobiology,* **65,** 77-89.

Roitblat, H., Tham, W. & Golub, L. (1982) Performance ofBetta splendens in a radial arm maze. *Animal Learning & Behavior,* **10,** 108-114.

Sanchez-Bayo, F. & Goka, K. (2014) Pesticide Residues and Bees – A Risk Assessment. *PloS one,* **9,** e94482.

Sarkar, D. (2008) *Lattice: Multivariate Data Visualization with R.* Springer, New York.

Skaug, H., Fournier, D., Bolker, B., Magnusson, A. & Nielsen, A. (2014) _Generalized Linear Mixed Models using AD Model Builder_.

Stanley, D.A., Smith, K.E. & Raine, N.E. (2015) Bumblebee learning and memory is impaired by chronic exposure to a neonicotinoid pesticide. *Scientific reports,* **5.**

Sulikowski, D. & Burke, D. (2010) Reward type influences performance and search structure of an omnivorous bird in an open-field maze. *Behavioural processes,* **83,** 31-35.

Therneau, T. (2015) _A Package for Survival Analysis in S_.

Thompson, H., Harrington, P., Wilkins, S., Pietravalle, S., Sweet, D. & Jones, A. (2013) Effects of neonicotinoid seed treatments on bumble bee colonies under field conditions. *FERA Report*, pp. 76. FERA, York.

**a**	Correct choices in first eight choices		
Model	AIC	ΔAIC	w_i
**Basic**	**542**	**0**	**0.525**
**Size**	**543**	**0.92**	**0.331**
Treatment	545.6	3.52	0.09
Treatment + size	546.7	4.66	0.051
Treatment * size	552.1	10.09	0.003
**b**	Time per visit		
Model	AIC	ΔAIC	w_i
**Size**	**15**	**0**	**0.913**
Basic	20.2	5.26	0.066
Treatment + size	22.7	7.71	0.019
Treatment * size	28.3	13.34	0.001
Treatment	32	17.06	0

Table S1. Tables of candidate models to investigate the effect of pesticide treatment and bee size on a) correct choices in first eight choices using binomial GLMMs and b) duration divided by total visits using linear mixed models. The basic model included the constant and the residual variance, with all other models containing the basic model plus the indicated covariates. Models are presented in order of ΔAIC from the best model alongside their respective Akaike weights (w_i). The best set of models (models <2 ΔAIC from the model with the lowest AIC) is highlighted in bold.

**a**	Total revisits		
Model	AIC	ΔAIC	w_i
**Treatment**	**285.3**	**0**	**0.303**
**Treatment + Size**	**285.7**	**0.3**	**0.256**
**Size**	**286.1**	**0.7**	**0.209**
**Basic**	**286.3**	**1.0**	**0.186**
Treatment * Size	289.1	3.8	0.046
**b**	Choices before first revisit		
Model	AIC	ΔAIC	w_i
**Treatment**	**273**	**0**	**0.474**
Treatment + Size	275	2	0.174
Size	275.5	2.5	0.136
Basic	275.6	2.6	0.129
Treatment * Size	276.4	3.4	0.087
**c**	Time per visit		
Model	AIC	ΔAIC	w_i
**Size**	**13**	**0**	**0.75**
Basic	15.6	2.55	0.209
Treatment + Size	19.3	6.32	0.032
Treatment * Size	22.3	9.33	0.007
Treatment	24.6	11.55	0.002

Table S2. Analysis including field-realistic doses only of the effect of pesticide on RAM performance measures. Tables show candidate models a) using negative binomial GLMMs to investigate the effect of pesticide treatment and bee size on total revisits, b) using Cox proportional hazards models to investigate the effect of pesticide treatment and bee size on choices before first revisit and c) using linear mixed models to investigate the effect of pesticide treatment and bee size on log-transformed duration divided by total visits. In all cases, the basic model included the constant and the residual variance, with all other models containing the basic model plus the indicated covariates. Models are presented in order of ΔAIC from the best model alongside their respective Akaike weights (wi). The best sets of models which were averaged to obtain model averaged estimates (models <2 ΔAIC from the model with the lowest AIC) are highlighted in bold.

a	Total revisits			
Parameters	Estimate	Std. Error	95% CIs	
			Lower	Upper
(Intercept)	3.167	1.513	0.202	6.132
Treatment (LD.377)	0.344	0.205	-0.058	0.745
**Treatment (LD.091)**	**0.417**	**0.199**	**0.027**	**0.807**
Size	-0.358	0.259	-0.865	0.150

b	Correct choices before first revisit				
Parameters	Regression coefficient (b)	Std. Error (SE(b))	Hazard Ratio (e^b)	95% CIs on Hazard Ratio	
				Lower	Upper
Treatment (LD.377)	0.295	0.366	1.343	0.656	2.751
**Treatment (LD.091)**	**0.823**	**0.382**	**2.276**	**1.077**	**4.810**

c	Time per visit			
Parameters	Estimate	Std. Error	95% CIs	
			Lower	Upper
(Intercept)	1.563	0.643	0.302	2.824
**Size**	**-0.323**	**0.118**	**-0.555**	**-0.091**

Table S3. Analysis including field-realistic doses only of the effect of pesticide on RAM performance measures. Tables show model averaged coefficients and 95% confidence intervals (CIs) for the optimal model set to predict a) total revisits (treatment, treatment + size, size only and basic models with colony as a random effect), b) correct choices before first revisit (treatment only model) and c) time per visit (duration divided by total visits; size only model with colony as a random effect). Parameters highlighted in bold are considered important to the model based on 95% CIs.

a

Previous location	Destination flower 1	2	3	4	5	6	7	8
Tunnel	13%	19%	16%	19%	3%	13%	3%	13%
1	5%	19%	16%	5%	5%	16%	19%	14%
2	19%	3%	15%	9%	8%	10%	18%	17%
3	6%	37%	6%	13%	6%	12%	9%	10%
4	0%	10%	35%	2%	15%	18%	17%	2%
5	2%	8%	10%	36%	0%	24%	19%	1%
6	2%	3%	8%	8%	31%	3%	38%	9%
7	8%	8%	5%	8%	16%	13%	2%	42%
8	24%	9%	12%	13%	10%	10%	17%	5%
Total % visits to:	9%	13%	14%	12%	11%	13%	16%	13%

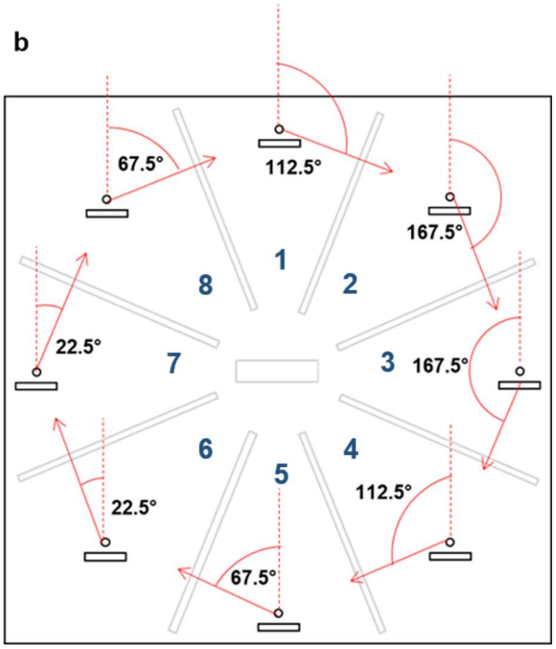

Figure S1. a) Global transition probability matrix showing probabilities of moving from the tunnel (arena entrance) or a flower to each other possible flower. This was used to construct a Monte Carlo simulation of performance on the radial arm maze (RAM) using stereotypical behaviour only. b) Schematic of the RAM with the angle from vertical of each transition between neighbouring flowers. Angle from vertical was negatively correlated with transition probability (Spearman's rho = -0.87).

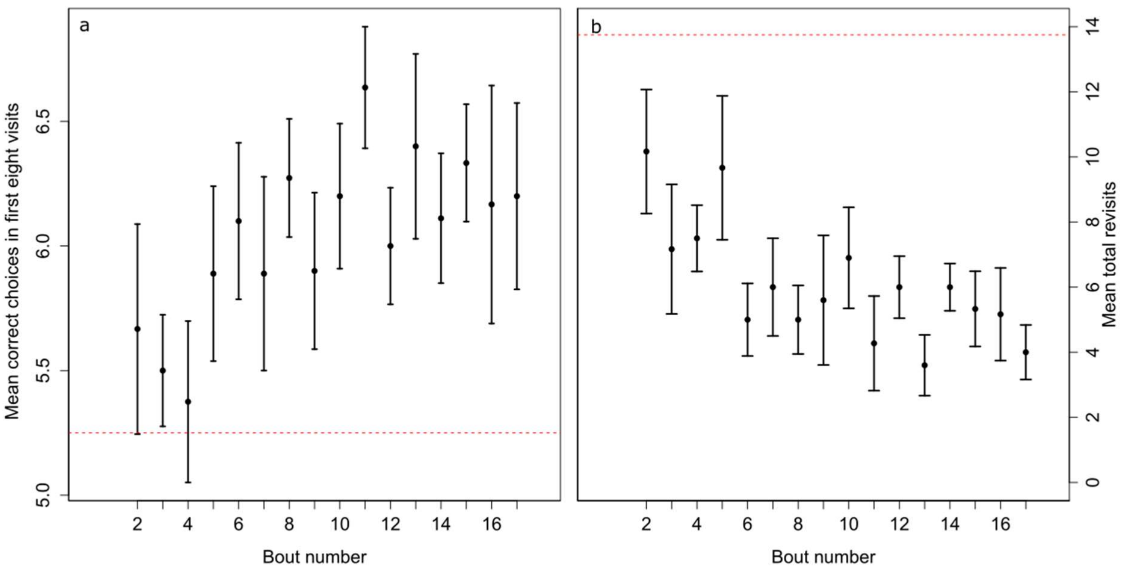

Figure S2. Data from a pilot study using 14 bees showing a) mean correct choices in first eight choices (±SE) and b) mean total revisits (±SE) by bout number. Expected chance performance for both measures is indicated by a red dashed line.

Figure S3. *(Below)* Outputs of two Monte Carlo simulations run with 1,000,000 iterations simulating a) pure chance (C) and b) chance plus stereotypical behaviour (C+S), showing frequency tables and means for three measures of RAM performance: correct choices in first eight choices, correct choices before first revisit and total revisits. The frequency tables for total revisits are truncated at 75 revisits, although the actual data from which the means are calculated include values greater than 75. Mean total revisits with revisits capped at 18 is also shown to account for physical limitations on maximum number of revisits in the observed data. The simulations were coded using C++.

Fig. S3 cont.

a) Chance

Mean total revisits

Total revisits	Frequency
0	2440
1	8565
2	17274
3	27222
4	37588
5	46498
6	52243
7	56392
8	58477
9	58648
10	58021
11	55353
12	52128
13	48313
14	44875
15	41354
16	37258
17	33944
18	30152
19	27091
20	24379
21	21657
22	19033
23	17094
24	15037
25	13217
26	11703
27	10420
28	9009
29	7976
30	6876
31	6327
32	5438
33	4666
34	4123
35	3666
36	3159
37	2745
38	2497
39	2162
40	1887
41	1684
42	1418
43	1254
44	1023
45	922
46	846
47	777
48	710
49	586
50	503
51	453
52	381
53	303
54	267
55	240
56	217
57	198
58	160
59	147
60	120
61	114
62	95
63	75
64	88
65	57
66	56
67	54
68	42
69	38
70	37
71	28
72	15
73	28
74	15
75	13

b) Chance + stereotypical behaviour

Mean total revisits

Total revisits	Frequency
0	2791
1	9202
2	17527
3	26408
4	34862
5	41101
6	46002
7	48663
8	50387
9	50541
10	49129
11	47999
12	45453
13	42794
14	40797
15	37913
16	34899
17	32403
18	29809
19	27403
20	24663
21	22993
22	20865
23	19027
24	17081
25	15801
26	14321
27	12811
28	11901
29	10642
30	9948
31	8940
32	8235
33	7399
34	6695
35	6144
36	5649
37	5181
38	4696
39	4208
40	3875
41	3595
42	3268
43	2938
44	2740
45	2474
46	2350
47	2053
48	1900
49	1767
50	1593
51	1467
52	1395
53	1298
54	1098
55	1102
56	936
57	882
58	783
59	763
60	692
61	597
62	598
63	550
64	472
65	437
66	409
67	376
68	351
69	325
70	302
71	262
72	247
73	224
74	214
75	205

Fig. S3 cont.

a) Chance

```
Mean choice accuracy
Choice accuracy          Frequency
    1                    0
    2                    463
    3                    19134
    4                    170805
    5                    420407
    6                    319433
    7                    67318
    8                    2440

Mean choices before first revisit
Choices                  Frequency
    1                    125031
    2                    218360
    3                    245646
    4                    205829
    5                    128236
    6                    57586
    7                    16872
    8                    2440

Mean choice accuracy: 5.25093
Mean choices before first revisit: 3.24633
Mean total revisits: 13.7449
Mean total revisits (capped at 18): 9.93634
23.3255% of bees screened out
```

b) Chance + stereotypical behaviour

```
Mean choice accuracy
Choice accuracy          Frequency
    1                    0
    2                    424
    3                    17689
    4                    157348
    5                    409343
    6                    337005
    7                    75400
    8                    2791

Mean choices before first revisit
Choices                  Frequency
    1                    36932
    2                    199422
    3                    271827
    4                    243194
    5                    155058
    6                    70588
    7                    20188
    8                    2791

Mean choice accuracy: 5.30218
Mean choices before first revisit: 3.5865
Mean total revisits: 16.064
Mean total revisits (capped at 18): 9.94618
31.132% of bees screened out
```
